# Parental occupational exposure to solvents and risk of developing testicular germ cell tumors among sons: a French nationwide case-control study (TESTIS study)

**DOI:** 10.5271/sjweh.4102

**Published:** 2023-09-01

**Authors:** Margot Guth, Marie Lefevre, Corinne Pilorget, Astrid Coste, Shukrullah Ahmadi, Aurélie Danjou, Brigitte Dananché, Delphine Praud, Isabelle Koscinski, Aline Papaxanthos, Oxana Blagosklonov, Patricia Fauque, Olivia Pérol, Joachim Schüz, Louis Bujan, Ann Olsson, Béatrice Fervers, Barbara Charbotel, the TESTIS study group

**Affiliations:** 1 UMRESTTE, UMR T 9405, IFSTTAR, Lyon 1 University, Lyon University, Eiffel University, Lyon, France.; 2 Santé publique France - French National Public Health Agency, Saint-Maurice, France.; 3 INSERM UMR1296 Radiation: Defense, Health, Environment, Lyon, France.; 4 Prevention Cancer Environnement Departement, Centre Léon Bérard, Lyon, France.; 5 Environment and Lifestyle Epidemiology Branch, International Agency for Research on Cancer/World Health Organization (IARC/WHO), Lyon, France.; 6 Laboratory of Biology of Reproduction-CECOS Lorraine, University Hospital of Nancy, Nancy, France.; 7 Reproductive Medicine and Biology Department, Centre Hospitalier Universitaire de Bordeaux, Bordeaux, Aquitaine, France.; 8 CECOS Franche-Comté Bourgogne Besançon, CHU de Besançon, Besançon, France.; 9 University of Bourgogne Franche-Comté - INSERM UMR1231, Dijon, France.; 10 Laboratory of Biology of Reproduction-CECOS, University Hospital of Dijon, Dijon, France.; 11 DEFE (Développement Embryonnaire, Fertilité, Environnement) INSERM 1202 Universités Montpellier et Toulouse 3, CECOS Hôpital Paule de Viguier, CHU de Toulouse, Toulouse, France.; 12 Fédération Française des CECOS, Paris, France.; 13 CRPPE-Lyon, Hospices Civils de Lyon, Lyon, France.

**Keywords:** cancer, epidemiology, JEM, job-exposure matrix, organic solvent, prenatal exposure, testicular cancer

## Abstract

**Objectives:**

The etiology of testicular germ cell tumors (TGCT) is suspected to be related to prenatal environmental risk factors. Some solvents have potential endocrine disrupting or carcinogenic properties and may disrupt male genital development in utero. The aim of this study was to examine the association between parental occupational exposure to solvents and TGCT risk among their offspring.

**Methods:**

A French nationwide case–control study, TESTIS included 454 TGCT cases and 670 controls frequency-matched on region and 5-year age strata. Participants were interviewed via telephone and provided information on parental occupations at birth. Job-exposure matrices (JEM) developed in the French Matgéné program were used to assign exposure to five petroleum-based solvents, five solvents or groups of oxygenated solvents, and five chlorinated solvents. Odds ratios (OR) for TGCT and 95% confidence intervals (CI) were estimated using conditional logistic regression, adjusting for TGCT risk factors.

**Results:**

Occupational exposure to at least one solvent during the year of their son’s birth was 41% among fathers and 21% among mothers. Paternal exposure to at least one solvent showed OR 0.89 (95% CI 0.68–1.15). Exposure to perchloroethylene (OR 1.41, 95% CI 0.55–3.61), methylene chloride (OR 1.13, 95% CI 0.54–2.34) and diesel/kerosene/fuel oil (OR 1.17, 95% CI 0.80–1.73) disclosed OR >1 but with low precision. Our results suggest a possible modest increase in non-seminoma risk for sons whose fathers were highly exposed to trichloroethylene (OR 1.44, 95% CI 0.79–2.63). Maternal exposure to at least one solvent showed OR 0.90 (95% CI 0.65–1.24). When stratifying by birth year, men born in the 1970s experienced an increased TGCT risk following maternal exposure to fuels and petroleum-based solvents (OR 2.74, 95% CI 1.11–6.76).

**Conclusion:**

Overall, no solid association was found between parental occupational exposure to solvents and TGCT risk. The association found with maternal occupational exposure to fuels and petroleum solvents among older men needs further investigation.

Testicular cancer is the most common cancer affecting Caucasian men, aged 15–44 years (1–3). Most cases (>95%) are germ cell tumors (TGCT), the majority of which derive from germ cell neoplasia in situ (GCNIS-TGCT) developed in utero and are subdivided into two main histological subtypes: seminoma and non-seminoma (3, 4). GCNIS-TGCT incidence has increased in recent decades and continues to rise (2, 5). In light of the rapid incidence increase, a contribution of environmental and lifestyle factors to the increased risk of TGCT is suspected (4), although the precise causal factors remain largely unknown (6). The impact of the fetal environment is also supported by large geographical variation in incidence (7) and the evolution of TGCT incidence among migrant populations (8).

TGCT has been suggested to be part of testicular dysgenesis syndrome (TDS), which includes other male reproductive system disorders, ie, cryptorchidism, hypospadias and poor semen quality (9). TDS could be a consequence of intrauterine exposure to environmental compounds with endocrine disrupting properties, which disrupt the physiological actions of endogenous hormones during the fetal "masculinization programming window (MPW)" (3, 6, 8), and stop the development of immature germ cells that persist outside of fetal/perinatal life (CGNIS) (10).

Many workers are exposed to solvents that are widely used in a range of industrial products (11). Some are suspected of acting as endocrine disrupting chemicals (EDC) (12) and may interfere in the masculinization process in utero (13) or are classified as carcinogenic by the International Agency for Research on Cancer (IARC) monographs (14). In addition, in the 2000s, about one-third of the industrial solvents tested were considered reproductive toxicants in laboratory animals by inhalation and dermal routes, suggesting that they may have a high potential for reproductive toxicity in humans by the same exposure routes (15, 16). Experimental studies have recently suggested that exposure to trichloroethylene (TCE) may induce epigenetic alterations (17, 18), which may result in developmental disorders (19).

Epidemiological studies suggest that maternal exposure to some EDC during pregnancy is associated with birth urogenital anomalies, but observations of a link to the adverse adult male reproductive health, such as TGCT, are inconsistent (20). In addition, only few previous studies have examined parental exposures to solvents and TGCT in their offspring. A registry-based case–control study in Finland, Norway, and Sweden (NORD-TEST) suggested an increase in TGCT risk associated with maternal occupational exposure to toluene, but none for paternal exposure, except for tetrachloroethylene [or perchloroethylene (PCE)] exposure in Finland (21). The Denmark NORD-TEST study and a US case–control study found no association between parental solvent exposure and TGCT among sons (22, 23). These studies had limitations related to the registry-based nature of the data (ie, missing information of potential cofounders and delay between the child’s birth and the nearest census to assess occupational exposure) or the small numbers of cases.

This study aims to examine the associations between parental occupational exposure to solvents and the risk of developing TGCT in the sons in a French national case–control study.

## Methods

### Study population

The TESTIS study (24) is a multicenter prospective case–control study. Cases of histologically confirmed TGCT (aged 18–45 years), referred to a CECOS (*Centres d’étude et de conservation des oeufs et du sperme /* Center for the Study and Conservation of Eggs and Sperm) for sperm cryopreservation prior to treatment, were identified between January 2015 and April 2018. A TGCT expert reviewed the cases and confirmed GCNIS-related TGCT cases based on pathology reports and serum tumor markers and classified them into seminoma and non-seminomatous tumors (3, 25).

Two control groups with no personal history of testicular cancer or cryptorchidism were frequency-matched to cases on year of birth (+/-5 years) and hospital center’s region: Group A controls – sperm donors with normal sperm production and partners of women consulting for fertility disorders in CECOS; Group B controls – partners of women treated for a pathological pregnancy in specialized maternity clinics adjacent to the CECOS.

Detailed information on study population and reasons for non-participation has been reported previously (24). Briefly, a total of 1463 eligible subjects were invited to participate in the study. Of these, 1367 participants (93.4%) agreed to participate, and 96 cases and 147 controls were excluded for various reasons, including non-GCNIS TGCT, incomplete telephone interview, or not born in "metropolitan France". Thus, 1124 participants were included in the analyses (supplementary material, www.sjweh.fi/article/4102, figure S1).

### Data collection

A trained investigator (IPSOS company) conducted a telephone interview where participants provided information on residential history since birth, characteristics at birth, medical history, lifestyle factors (smoking and drug use), and parental employment at year of birth. The telephone interview was conducted using a structured, pretested, computer-assisted questionnaire, and the interviewers were blinded to case–control status. Moreover, participants received on inclusion written support to prepare for the interview.

### Exposure assessment

An industrial hygienist coded parental occupations according to the International Standard Classification of Occupation, 1968 version (ISCO-68) (26) and industries according to the *Nomenclature d’activités française* 1999 version (French classification of economic activities NAF-99, updated version of NAF-93) (27). Job-exposures matrices (JEM) developed for the French population in the context of the Matgéné program (28) were used to assess occupational exposures to: (i) five solvents or groups of oxygenated solvents (alcohols; ketones and esters; ethylene glycol; diethyl ether; tetrahydrofuran); (ii) five chlorinated solvents [TCE; PCE; methylene chloride (MC); carbon tetrachloride; chloroform]; and (iii) five fuels & petroleum-based solvents (benzene; automobile gasoline; white spirits and other aromatics; diesel, kerosene and fuel oil (KDF); special petroleum products and other aliphatics).

For each parental job at birth, defined as a combination of ISCO and NAF codes, the JEM provided three indices of exposure for each individual solvent: probability of exposure (P), expressed as the percentage of exposed workers; intensity of exposure (I), expressed in parts per million (ppm) or according to a semi-quantitative scale; and frequency of exposure (F), expressed as a percentage of working time with solvent exposure. For exposure to at least one solvent within the same family, the JEM provided an average level (L) of exposure during a usual working day, combining intensity, and frequency indices. To account for changes in exposure over this period, indices have been provided for different calendar periods (eg, periods defined for TCE exposure are: 1950–1969; 1970–1984; 1985–1994, 1995–2012 and 2013–2016). Each of the four indices (P, I, F, L) was divided into different classes of values, which may vary depending on the solvent considered (supplementary table S1). Some jobs could not be coded due to insufficient information or lack of details and were therefore considered as missing data (N=36 mothers’ jobs, N=47 fathers’ jobs). Unemployed, students, and military jobs were treated as unexposed, unless additional information was available on student or military employment.

Finally, parental occupational exposure estimates (PEE) were calculated as the product of exposure P, F, I, using the central value of the classes (weight table S1). For exposure to at least one solvent within the same family, L was used in PEE calculation (P×L). Each PEE was classified in three categories: "unexposed" (referent group), "low exposed" and "high exposed". The cutoffs of exposure were specific to each solvent based on the distribution of PEE among fathers and mothers of controls and were set at the 50^th^ percentile (supplementary table S2). Models were built using the unexposed group of each solvent individually as the reference category.

Because exposure to one solvent does not preclude exposure to others, exposure to a given solvent was also categorized as a three-category exposure using a method previously applied (21): "unexposed to any solvents" (referent group), "exposed to the specific solvent" (with or without exposure to other solvents) and "exposed only to solvent(s) other than the specific solvent".

### Statistical methods

Odds ratios (OR) and corresponding 95% confidence intervals (CI) for TGCT in adulthood were estimated for parental occupational exposure to solvents, using conditional logistic regression models. All models were conditioned for matching factors (region and birth year grouped in 5-year categories). Univariate analysis was used to assess associations of TGCT with literature-based considered factors (29–31). These included: birth weight, gestational age, birth order, sibship size and born from multiple pregnancy, family history of testicular cancer, family history of cryptorchidism, personal tobacco smoking (32), personal consumption of alcoholic beverages (33) and personal history of testicular trauma (30). As suggested by Hodes-Simeon et al (34), we also used 'age at voice change' as a proxy of the timing of pubertal development to consider delayed puberty, which is a protective factor for TGCT. We selected those with P<0.20. All selected covariates were then included in one single regression model, and a manual backward stepwise selection procedure was performed. The final model included the following variables significantly associated with TGCT (P<0.05): sibship size, being born from multiple pregnancy, personal history of testicular trauma, family history of TGCT, and family history of cryptorchidism.

Spearman correlation coefficients were computed to assess pair-wise correlations between solvent exposures. To consider of multicollinearity between solvents, a principal component analysis (PCA) was also used to confirm possible association between solvents’ exposure and TGCT risk. Only the PC whose eigenvalue was superior to 1 were retained. The association between the PC scores and TGCT risk was then estimated using conditional logistic regression analysis. Main analyses were repeated for histological subtype of tumors and the heterogeneity of associations was tested using polytomous logistic regression for matched case–control studies (SAS macro %subtype) (35). P-values for heterogeneity were derived from the likelihood ratio test (35).

Given the identified toxicity of some solvents, such as benzene and TCE, regulations concerning their uses have largely evolved over time, suggesting that exposures levels may have decreased in recent decades (28). Thus, the analysis was stratified by birth cohort (1969–1980; 1981–1990; 1991–1999). The Wald test was used to assess homogeneity across birth years (36).

Additionally, we conducted several sensitivity analyses: we excluded cases with a personal history of cryptorchidism (N=40) and cases not confirmed by pathology reports (N=43). Given the broad age strata (5-year categories), we further adjusted for age to avoid residual confounding by age in birth age strata.

Data analysis was performed using SAS statistical software version 9.4 (SAS Institute Inc, Cary, NC, USA). In all statistical analyses, P-values were two-sided and considered statistically significant if <0.05.

## Results

The main characteristics of TGCT cases and controls are shown in table 1. Cases [mean 31.9 (SD 6.1) years] were younger than controls [33.6 (SD 5.4) years] (P<0.001). Cases were more often born from multiple pregnancies (P=0.02), first born (P=0.04). Also, more cases than controls reported personal history of: inguinal hernia (P=0.01), testicular trauma (P=0.004), family history of TGCT (P=0.001), and cryptorchidism (P=0.01). Cases reported also greater alcohol consumption (P=0.03) than controls. Finally, there was no difference between cases and both control groups in 'age at voice change', smoking status, cannabis use, birth weight or gestational age.

**Table 1:** Characteristics of testicular germ cell tumors (TGCT) cases and controls in the TESTIS study, 2015–2018 (N=1124).

Characteristics		TGCT cases (N=454)		Controls (N=670)	P-value cases / all controls*
		N (%)		N (%)	
Age at diagnosis (years)					<0.001
	≤25	64 (14.1)		32 (4.8)	
	26–30	106 (23.4)		144 (21.5)	
	31–35	113 (24.9)		239 (35.7)	
	36–40	85 (18.7)		162 (24.2)	
	≥41	43 (9.5)		93 (13.9)	
	Missing	43 (9.5)		0 (0.0)	
Year of birth					<0.001
	Before 1975	37 (8.2)		50 (7.5)	
	1975–1979	80 (17.6)		128 (19.1)	
	1980–1984	117 (25.8)		218 (32.5)	
	1985–1989	130 (28.6)		203 (30.3)	
	1990–1994	70 (15.4)		63 (9.4)	
	1995–1999	20 (4.4)		8 (1.2)	
Education					0.10
	Secondary	180 (39.7)		203 (30.3)	
	1–2-years university degree	97 (21.4)		148 (22.1)	
	>3 years university degree	128 (28.2)		234 (34.9)	
	Other	48 (10.6)		85 (12.7)	
	Missing	1 (0.2)		0 (0.0)	
Smoking status					0.12
	Former smoker	102 (22.5)		171 (25.5)	
	Current smoker	147 (32.4)		185 (27.6)	
	Never smoker	205 (45.2)		314 (46.9)	
Birth weight (g)					0.49
	<2500	25 (5.5)		33 (4.9)	
	2500–4000	356 (78.4)		561 (83.7)	
	≥4000	46 (10.1)		58 (8.7)	
	Missing	27 (6.0)		18 (2.7)	
Gestational age (weeks)					0.16
	≤36	32 (7.1)		34 (5.1)	
	>36	415 (91.4)		629 (93.9)	
	Missing	7 (1.5)		7 (1.0)	
Born from multiple pregnancy					0.02
	No	433 (95.4)		652 (97.3)	
	Yes	21 (4.6)		17 (2.5)	
	Missing	0 (0.0)		1 (0.2)	
Birth order					0.04
	First	213 (46.9)		305 (45.5)	
	Second	163 (35.9)		224 (33.4)	
	Third	63 (13.9)		93 (13.9)	
	Fourth or more	15 (3.3)		48 (7.2)	
Sibship size					0.03
	1	25 (5.5)		59 (8.8)	
	2	192 (42.3)		254 (37.9)	
	3	153 (33.7)		206 (30.8)	
	≥4	84 (18.5)		150 (22.4)	
	Missing	0 (0.0)		1 (0.2)	
Personal history of inguinal hernia					0.01
	No	414 (91.2)		637 (95.1)	
	Yes	39 (8.6)		32 (4.8)	
	Missing	1 (0.2)		1 (0.2)	
Personnal history of testicular trauma					0.004
	No	387 (85.2)		611 (91.2)	
	Yes	67 (14.8)		59 (8.8)	
Family history of TGCT					< 0.001
	No	419 (92.3)		651 (97.2)	
	Yes	33 (7.3)		16 (2.4)	
	Missing	2 (0.4)		3 (0.4)	
Family history of cryptorchidism					0.01
	No	422 (93)		645 (96.3)	
	Yes	28 (6.2)		18 (2.7)	
	Missing	4 (0.9)		7 (1.0)	
Age at voice change (years)					0.61
	<12	16 (3.5)		26 (3.9)	
	12–16	375 (82.6)		532 (79.4)	
	>16	53 (11.7)		94 (14.0)	
	Missing	10 (2.2)		18 (2.7)	
Cannabis use (12–17 years)					0.30
	No	312 (68.7)		443 (66.1)	
	Yes	142 (31.3)		227 (33.9)	
Frequency of cannabis use (12–17 years)					0.24
	Never	312 (68.72)		443 (66.1)	
	<Once / month	51 (11.23)		84 (12.5)	
	≥Once / month	22 (4.85)		47 (7.0)	
	Once / week	37 (8.15)		39 (5.8)	
	Once / day	32 (7.05)		57 (8.5)	
Cannabis use (18–25 years)					0.68
	No	262 (57.71)		393 (58.7)	
	Yes	192 (42.29)		276 (41.2)	
	Missing	0 (0.0)		1 (0.2)	
Frequency of cannabis use (18–25 years)					0.09
	Never	262 (57.7)		393 (58.7)	
	<Once / month	62 (13.7)		107 (16.0)	
	≥1 time / month	23 (5.1)		46 (6.9)	
	Once / week	41 (9.0)		54 (8.1)	
	Once / day	66 (14.5)		69 (10.3)	
	Missing	0 (0.0)		1 (0.2)	
Paternal occupation at childbirth (ISCO-68)					0.09
	Professionnal, technical & related workers	91 (20.0)		175 (26.1)	
	Administration and managerial workers)	21 (4.6)		41 (6.1)	
	Clerical and related workers	37 (8 .2)		53 (7.9)	
	Sales workers	35 (7.7)		42 (6.3)	
	Service workers	38 (8.4)		33 (4.9)	
	Agricultural, animal husbandry and forestry workers, fishermen and hunters	32 (7.1)		37 (5.5)	
	Production and related workers, transport equipment operators and laborers	164 (36.1)		234 (34.9)	
	Unemployed	11 (2.4)		24 (3.6)	
	Military	1 (0.2)		8 (1.2)	
	Missing	24 (5.3)		11 (2.4)	
Maternal occupation at childbirth (ISCO-68)					0.66
	Profesonnal, technical & related workers	88 (19.4)		137 (20.5)	
	Administration and managerial workers	6 (1.3)		11 (1.6)	
	Clerical and related workers	97 (21.4)		125 (18.7)	
	Sales workers	24 (5.3)		35 (5.2)	
	Service workers	47 (10.4)		75 (11.2)	
	Agricultural, animal husbandry and forestry workers, fishermen and hunters	10 (2.2)		14 (2.1)	
	Production and related workers, transport equipment operators and laborers	19 (4.2)		46 (6.9)	
	Unemployed	145 (31.9)		209 (31.2)	
	Missing	18 (4.0)		18 (4.0)	
Paternal industry at childbirth (NAF-99)					
	Agriculture,hunting and forestry	32 (7.1)		38 (5.7)	0.87
	Fishing	1 (0.2)		0 (0.0)	
	Mining and quarrying	1 (0.2)		3 (0.5)	
	Manufacturing	61 (13.4)		105 (15.7)	
	Electricity, gas and water supply	5 (1.1)		5 (0.8)	
	Construction	48 (10.6)		69 (10.3)	
	Wholsale and retail trade; *repair of motor vehicles, motorcycles and personal and household goods	50 (11.0)		62 (9.3)	
	Hotels and restaurants	11 (2.4)		13 (1.9)	
	Transport,storage and communication	44 (10.0)		60 (9.0)	
	Financial intermediation	11 (2.4)		19 (2.8)	
	Real estate, renting and business activities	21 (4.6)		43 (6.4)	
	Public administration and defence; *compulsory social security	41 (9.0)		45 (6.7)	
	Education	17 (3.7)		33 (4.9)	
	Health and social work	21 (4.6)		41 (6.1)	
	Other community, social and personal services activities	7 (1.5)		13 (1.9)	
	Private households with employed persons	0 (0.0)		0 (0.0)	
	Extra-territorial organizations and bodies	0 (0.0)		1 (0.2)	
	Unemployed	11 (2.4)		22 (3.3)	
	Missing or unknown	72 (15.9)		98 (14.6)	
	Maternal industry at childbirth (NAF-99)				0.97
	Agriculture, hunting and forestry	10 (2.2)		14 (2.2)	
	Fishing	0 (0.0)		0 (0.0)	
	Mining and quarrying	0 (0.0)		0 (0.0)	
	Manufacturing	21 (4.6)		43 (6.4)	
	Electricity, gas and water supply	0 (0.0)		2 (0.3)	
	Construction	0 (0.0)		1 (0.2)	
	Wholsale and retail trade; *repair of motor vehicles,motorcycles and personal and household goods	27 (6.0)		44 (6.6)	
	Hotels and restaurants	10 (2.2)		19 (2.8)	
	Transport, storage and communication	9 (2.0)		14 (2.1)	
	Financial intermediation	6 (1.3)		15 (2.2)	
	Real estate, renting and business activities	15 (3.3)		19 (2.8)	
	Public administration and defence; *compulsory social security	18 (4.0)		34 (5.1)	
	Education	37 (8.2)		49 (7.3)	
	Health and social work	56 (12.3)		93 (13.9)	
	Other community, social and personal services activities	9 (2.0)		14 (2.1)	
	Private households with employed persons	5 (1.1)		10 (1.5)	
	Extra-territorial organizations and bodies	0 (0.0)		0 (0.0)	
	Unemployed	145 (91.9)		206 (30.9)	
	Missing or unknown	86 (18.9)		92 (13.7)	

*P-values from bivariate conditional logistic regression models conditioned on region and birth year– except for the year of birth which was a matching factor.

About 67% of mothers and 97% of fathers were professionally active in the year of their son’s birth. Supplementary table S3 shows the most held occupation titles among fathers and mothers according to PEE of exposure to oxygenated, chlorinated and petroleum-based solvents.

### Correlations between exposures to solvents

Spearman correlations coefficients between solvent exposures ranged from r=-0.03 (between diethylether and TCE) to 1.00 (between ethylene glycol and automobile gasoline) for maternal exposures and from -0.05 (between kerosene/diesel oil/fuel oils and tetrahydrofurane) to 0.74 (between carbon tetrachloride and chloroform) for paternal exposures (supplementary table S4).

### Parental occupational exposures to solvents

The reported parental jobs were in the period 1969–1999. Approximately, 21% of the mothers and 41% of the fathers were occupationally exposed to at least one solvent during the year of their son’s birth (supplementary table S5). For the fathers’ and mothers’ jobs exposed to TCE, PCE, MC and benzene, most were assessed as exposed in the low/medium exposure level classes for an 8-hour workday (supplementary table S6).

Table 2 shows the adjusted OR (OR_adj_) for TGCT associated with PEE of organic solvents. For fathers the OR_adj_ for exposure to "any solvent" was 0.89 (95% CI 0.68–1.15). For KDF, the risk seems mainly driven by the low-exposure category (OR_adj_ 1.52, 95% CI 0.98–2.37). An inverse association with TGCT risk (OR_adj_ 0.71, 95% CI 0.52–0.97), and seminomas (OR_adj_ 0.75, 95% CI 0.57–0.97) was observed related to fathers’ exposure to oxygenated solvents. Among the chlorinted solvents, the low-exposure category to TCE was inversely associated with seminomas (OR_adj_ 0.45, 95% CI 0.23–0.90). Conversely, these analyses were also suggestive a positive association with non-seminomas among sons whose fathers were highly exposed to TCE (OR_adj_ 1.44, 95% CI 0.79–2.63). However, CI were wide and included the null hypothesis.

**Table 2:** Odds ratios (OR) and 95% confidence intervals (CI) for testicular germ cell tumors (TGCT) associated with paternal occupational exposure to solvents in the year of the child’s birth, overall and according to histological subtypes, TESTIS study, 2015–2018. [Ca/Co=Cases/controls; P-Het=P for heterogeneity].

Solvents exposure			All TGCT cases			Non-seminomas ^d^			Seminomas ^c^		P-Het^b^
			Ca/Co	OR (95% CI)^a^		Ca/Co	OR (95% CI)^a^		Ca/Co	OR (95% CI)^a^	
Any solvents											
	None		251/377	1.00		103/377	1.00		126/377	1.00	0.59
	Exposed to at least one solvent		162/264	0.89 (0.68–1.15)		67/264	0.85 (0.58–1.25)		75/264	0.74 (0.52–1.05)	
Oxygenated solvents											
	None		326/473	1.00		128/473	1.00		167/473	1.00	0.05
	Exposure		87/168	0.71 (0.52–0.97)		42/168	0.87 (0.67–1.15)		34/168	0.75 (0.57–0.97)	
		Low	47/84	0.74 (0.50–1.11)		27/84	1.17 (0.70–1.97)		14/84	0.44 (0.24–0.83)	
		High	40/84	0.68 (0.45–1.03)		15/84	0.66 (0.36–1.21)		20/84	0.67 (0.39–1.14)	
Alcohols											
	None		361/539	1.00		147/539	1.00		176/539	1.00	0.18
	Exposure		52/102	0.76 (0.52–1.11)		23/102	0.91 (0.54–1.53)		25/102	0.69 (0.42–1.13)	
		Low	25/51	0.68 (0.40–1.14)		13/51	1.09 (0.54–2.20)		9/51	0.44 (0.20–0.95)	
		High	27/51	0.85 (0.51–1.40)		10/51	0.75 (0.36–1.58)		16/51	0.97 (0.53–1.76)	
Ketones and esters											
	None		381/577	1.00		154/577	1.00		190/577	1.00	0.20
	Exposure		32/64	0.79 (0.50–1.25)		16/64	1.00 (0.55–1.83)		11/64	0.56 (0.28–1.11)	
		Low	20/44	0.72 (0.41–1.27)							
		High	12/20	0.94 (0.44–2.00)							
Ethylene glycol											
	None		404/624	1.00							
	Exposure		9/17	0.69 (0.29–1.61)							
		Low									
		High									
Chlorinated solvents											
	None		344/519	1.00		138/519	1.00		175/519	1.00	0.24
	Exposure		69/122	0.78 (0.55–1.10)		32/122	1.03 (0.77–1.38)		26/122	0.73 (0.54–0.99)	
		Low	31/66	0.63 (0.40–1.01)		15/66	0.77 (0.41–1.43)		11/66	0.43 (0.22–0.86)	
		High	38/56	0.96 (0.61–1.52)		17/56	1.21 (0.65–2.24)		15/56	0.68 (0.36–1.28)	
Trichloroethylene											
	None		346/525	1.00		138/525	1.00		176/525	1.00	0.14
	Exposure		67/116	0.80 (0.56–1.13)		32/116	1.03 (0.64–1.64)		25/116	0.54 (0.33–0.88)	
		Low	29/63	0.61 (0.38–1.00)		14/63	0.74 (0.39–1.41)		11/63	0.45 (0.23–0.90)	
		High	38/53	1.02 (0.65–1.63)		18/53	1.44 (0.79–2.63)		14/53	0.64 (0.33–1.23)	
Perchloroethylene											
	None		403/631	1.00							
	Exposure		10/10	1.41 (0.55–3.61)							
		Low									
		High									
Methylene chloride											
	None		399/622	1.00		165/622	1.00		195/622	1.00	0.82
	Exposure		14/19	1.13 (0.54–2.34)		5/19	1.07 (0.36–3.18)		6/19	0.91 (0.35–2.36)	
		Low	6/10	0.85 (0.29–2.47)							
		High	8/9	1.45 (0.54–3.89)							
Fuels & petroleum-based solvents											
	None		295/458	1.00		117/458	1.00		151/458	1.00	0.04
	Exposure		118/183	0.95 (0.71–1.27)		53/183	0.95 (0.72–1.25)		50/183	0.89 (0.69–1.13)	
		Low	72/105	1.07 (0.75–1.51)		40/105	1.42 (0.90–2.23)		23/105	0.64 (0.38–1.07)	
		High	46/78	0.81 (0.54–1.23)		13/78	0.66 (0.34–1.28)		27/78	0.89 (0.54–1.47)	
Benzene											
	None		396/610	1.00		162/610	1.00		195/610	1.00	0.26
	Exposure		17/31	0.85 (0.45–1.60)		8/31	1.08 (0.47–2.52)		6/31	0.53 (0.21–1.35)	
		Low	7/16	0.71 (0.28–1.84)							
		High	10/15	0.98 (0.42–2.28)							
Automobile gasoline											
	None		389/598	1.00		163/598	1.00		188/598	1.00	0.60
	Exposure		24/43	0.74 (0.43–1.28)		7/43	0.55 (0.23–1.32)		13/43	0.74 (0.37–1.47)	
		Low	14/22	0.83 (0.40–1.70)							
		High	10/21	0.65 (0.29–1.45)							
Special petroleum products											
	None		400/623	1.00		162/623	1.00		196/623	1.00	0.31
	Exposure		13/18	1.03 (0.48–2.20)		8/18	1.64 (0.65–4.12)		5/18	0.80 (0.28–2.26)	
		Low									
		High									
White spirits											
	None		336/500	1.00		133/500	1.00		174/500	1.00	0.10
	Exposure		77/141	0.82 (0.60–1.14)		37/141	1.06 (0.69–1.64)		27/141	0.53 (0.33–0.84)	
		Low	47/80	0.87 (0.58–1.30)		23/80	1.09 (0.64–1.85)		16/80	0.55 (0.30–1.01)	
		High	30/61	0.77 (0.48–1.24)		14/61	1.02 (0.53–1.95)		11/61	0.49 (0.25–0.97)	
Diesel, kerosene, fuel oil											
	None		355/565	1.00		147/565	1.00		173/565	1.00	0.67
	Exposure		58/76	1.17 (0.80–1.73)		23/76	1.24 (0.72–2.13)		28/76	1.06 (0.64–1.74)	
		Low	46/50	1.52 (0.98–2.37)							
		High	12/26	0.58 (0.28–1.23)							

^a^ Estimates obtained comparing TGCT cases to group A and group B controls combined and adjusted for sibship size, being born from multiple pregnancy, personal history of testicular trauma, family history of TGCT and family history of cryptorchidism. Analysis was restricted to subjects with no missing data for the adjustment variables (N=12). If the “Low”/”High” categories have less than 5 cases or controls, the categories are grouped into “None” or “All” if the number of subjects was sufficient, otherwise the line is shaded. ^b^ P-value for heterogeneity derived from the Likelihood Ratio Test, comparing seminoma versus non-seminoma tumours. ^c^ 219 cases of seminoma TGCT were present in the TESTIS study. ^d^ 191 cases of non-seminoma TGCT were present in the TESTIS study.

The OR_adj_ for maternal exposures to "any solvent" was 0.90 (95% CI 0.65–1.24) (table 3). Most OR_adj_ associated with maternal exposures were ≤1.0. When exposure was subdivided into low and high exposure categories, none of the maternal exposures were found to be associated with TGCT risk. The associations of TGCT with maternal exposures to solvents showed no heterogeneity between seminomas and non-seminomas.

**Table 3:** Odds ratios (OR) and 95% confidence intervals (CI) for testicular germ cell tumors (TGCT) associated with maternal occupational exposure to solvents in the year of the child’s birth, overall and according to histological subtypes, TESTIS study, 2015–2018. [Ca/Co=Cases/controls; P-Het=P for heterogeneity].

Solvents exposure			All TGCT cases			Non-Seminomas ^d^			Seminomas ^c^			P-Het ^b^
			Ca/Co	OR (95% CI)^a^		Ca/Co	OR (95% CI)^a^		Ca/Co	OR (95% CI)^a^		
Any solvents												
	None		341/509	1.00		139/509	1.00		170/509	1.00		0.89
	Exposed to at least one solvent		84/136	0.90 (0.65–1.24)		34/136	0.89 (0.57–1.40)		42/136	0.93 (0.62–1.40)		
Oxygenated solvents												
	None		348/518	1.00		140/518	1.00		176/518	1.00		0.69
	Exposure		77/127	0.87 (0.63–1.22)		33/127	0.94 (0.60–1.48)		36/127	0.83 (0.54–1.27)		
		Low	72/113	0.89 (0.63–1.26)								
		High	5/14	0.69 (0.24–1.98)								
Alcohols												
	None		352/524	1.00		141/524	1.00		179/524	1.00		0.52
	Exposure		73/121	0.86 (0.61–1.20)		32/121	0.96 (0.61–1.52)		33/121	0.78 (0.50–1.21)		
		Low										
		High										
Ketones and esters												
	None		410/619	1.00		167/619	1.00		205/619	1.00		0.81
	Exposure		15/26	0.99 (0.51–1.96)		6/26	0.94 (0.36–2.45)		7/26	0.80 (0.33–1.97)		
		Low										
		High										
Diethylether												
	None		392/588	1.00		159/588	1.00		197/588	1.00		0.77
	Exposure		33/57	0.93 (0.58–1.48)		14/57	1.02 (0.53–1.95)		15/57	0.89 (0.48–1.65)		
		Low	24/35	1.13 (0.64–1.97)								
		High	9/22	0.62 (0.27–1.44)								
Chlorinated solvents												
	None		413/622	1.00		168/622	1.00		206/622	1.00		0.77
	Exposure		12/23	0.88 (0.43–1.82)		5/23	1.03 (0.37–2.86)		6/23	0.83 (0.33–2.12)		
		Low										
		High										
Trichloroethylene												
	None		417/628	1.00								
	Exposure		8/17	0.77 (0.32–1.83)								
		Low										
		High										
Fuel & petroleum-based solvents												
	None		392/598	1.00		159/598	1.00		196/598	1.00		0.94
	Exposure		33/47	0.97 (0.60–1.57)		14/47	0.94 (0.49–1.82)		16/47	0.97 (0.52–1.81)		
		Low	25/35	0.97 (0.56–1.68)								
		High	8/12	0.99 (0.39–2.51)								
White spirits												
	None		399/604	1.00		160/604	1.00		202/604	1.00		0.39
	Exposure		26/41	0.85 (0.50–1.45)		13/41	1.02 (0.52–2.03)		10/41	0.66 (0.31–1.38)		
		Low	20/29	0.85 (0.46–1.56)								
		High	6/12	0.87 (0.32–2.42)								
Diesel, kerosene, fuel oil												
	None		418/637	1.00								
	Exposure		7/8	1.39 (0.48–4.07)								
		Low										
		High										

^a^ Estimates obtained comparing TGCT cases to group A and group B controls combined and adjusted for sibship size, being born from multiple pregnancy, personal history of testicular trauma, family history of TGCT and family history of cryptorchidism. Analysis was restricted to subjects with no missing data for the adjustment variables (N=12). If the “Low”/”High” categories have less than 5 cases or controls, the categories are grouped into “None” or “All” if the number of subjects was sufficient, otherwise the line is shaded. ^b^ P-value for heterogeneity derived from the Likelihood Ratio Test, comparing seminoma versus non-seminoma tumours. ^c^ 219 cases of seminoma TGCT were present in the TESTIS study. ^d^ 191 cases of non-seminoma TGCT were present in the TESTIS study.

Table 4 presents the results based on the second analytical approach where the reference category is mothers/fathers who have not been exposed to the solvents in the category of interest. Except for an inverse association observed for sons of fathers exposed to oxygenated solvents in the year of their birth (OR_adj_ 0.72, 95% CI 0.52–0.99), no associations between paternal and maternal exposures to solvents and TGCT risk was observed.

**Table 4:** Odds ratios (OR) and 95% confidence intervals (CI) for testicular germ cell tumors (TGCT) associated with parental occupational exposure to different solvents in the year of the child’s birth, with unexposed to any of the solvents as reference category, TESTIS study, 2015–2018. [Ca/Co=cases/controls]. Note: Exposed=exposed to the solvents of interest but can also be exposed to other solvents; Exposed only to others=exposed to any other solvents but the solvent of interest; Unexposed=unexposed to any of the individual or groups of solvents.

		Paternal exposure All TGCT cases			Maternal exposure All TGCT cases	
		Ca/Co	OR (95% CI) ^a^		Ca/Co	OR (95% CI) ^a^
Oxygenated solvents exposure						
	None	251/377	1.00		341/509	1.00
	Exposed	87/168	0.72 (0.52–0.99)		77/127	0.88 (0.63–1.22)
	Exposed only to other solvents	75/96	1.04 (0.72–1.49)		7/9	1.20 (0.43–3.35)
Chlorinated solvents exposure						
	None	251/377	1.00		341/509	1.00
	Exposed	69/122	0.76 (0.53–1.08)		12/23	0.86 (0.42–1.79)
	Exposed only to other solvents	93/142	0.90 (0.65–1.25)		72/113	0.90 (0.64–1.28)
Fuels & petroleum-based solvents exposure						
	None	251/377	1.00		341/509	1.00
	Exposed	118/183	0.90 (0.67–1.21)		33/47	0.95 (0.59–1.54)
	Exposed only to other solvents	44/81	0.70 (0.45–1.07)		51/89	0.87 (0.59–1.28)

^a^ Estimates obtained comparing TGCT cases to group A and group B controls combined and adjusted for sibship size, being born from multiple pregnancy, personal history of testicular trauma, family history of TGCT and family history of cryptorchidism. Analysis was restricted to subjects with no missing data for the adjustment variables (N=12). If the “Low”/”High” categories have less than 5 cases or controls, the categories are grouped into “None” or “All” if the number of subjects was sufficient, otherwise the line is shaded. ^b^ At least one of the following solvents: benzene; automobile gasoline; white spirits and other aromatics; diesel, kerosene and fuel oil; special petroleum products and other aliphatics; alcohols; ketones and esters; ethylene glycol; diethyl ether; tetrahydrofuran; trichloroethylene; perchloroethylene; methylene chloride; carbon tetrachloride; chloroform.

Depending on the exposure to the solvent of interest, variability in the OR_adj_ across different birth decades was observed (table 5). For paternal exposure, the OR_adj_ for oxygenated solvents exposure shows an inverse association in the 1970s (0.45, 95% CI 0.26–0.78), but an elevated OR_adj_ in the 90s (1.87; 95% CI 0.61–5.72), although the CI was wide. The associations between maternal occupational exposure to petroleum-based solvents and TGCT risk in sons varied by decade (P=0.01). A positive association was observed between mothers exposed to petroleum-based solvents in the 1969–1980 decades and TGCT risk in the offspring (OR_adj_ 2.74, 95% CI 1.11–6.76), but with a wide CI, and was not found among mothers exposed in the 1980s/90s. The other solvents did not appear to vary among son’s decade of birth.

**Table 5a:** Odds ratios (OR) and 95% confidence intervals (CI) for TGCT associated with **paternal** occupational exposure to different solvents in the year of the child’s birth, by period of birth year, TESTIS study, 2015–2018. [Ca/Co=cases/controls].

Solvents exposure		1969–80 (N=339)			1981–90 (N=612)			1991–99 (N=110)		P-value ^b^
		Ca/Co	OR (95% CI)^a^		Ca/Co	OR (95% CI)^a^		Ca/Co	OR (95% CI)^a^	
Oxygenated solvents exposure										
	Unexposed	98/142	1.00		186/292	1.00		46/39	1.00	0.09
	Exposed	27/72	0.45 (0.26–0.78)		46/87	0.85 (0.56–1.29)		14/9	1.87 (0.61–5.72)	
Chlorinated solvents exposure										
	Unexposed	104/164	1.00		195/316	1.00		45/39	1.00	0.67
	Exposed	21/50	0.62 (0.34–1.13)		37/63	0.97 (0.61–1.55)		11/9	0.88 (0.26–2.98)	
Fuels & petroleum-based solvents exposure										
	Unexposed	90/147	1.00		167/277	1.00		38/34	1.00	0.75
	Exposed	35/67	0.83 (0.49–1.39)		65/102	1.01 (0.68–1.48)		18/14	1.07 (0.40–2.89)	

^a^ Estimates obtained comparing TGCT cases to group A and group B controls combined and adjusted for sibship size, being born from multiple pregnancy, personal history of testicular trauma, family history of TGCT and family history of cryptorchidism. Analysis was restricted to subjects with no missing data for the adjustment variables (N=12). ^b^ If the categories of birth decades have less than 5 cases or controls, the categories “1981–90” and “1991–99” were combined into a single category (1981–99), otherwise the line is shaded.

**Table 5b:** Odds ratios (OR) and 95% confidence intervals (CI) for TGCT associated with **maternal** occupational exposure to different solvents in the year of the child’s birth, by period of birth year, TESTIS study, 2015–2018. [Ca/Co=cases/controls].

Solvents exposure		1969–80 (N=345)			1981–90 (N=614)			1991–99 (N=111)		P-value ^b^
		Ca/Co	OR (95% CI)^a^		Ca/Co	OR (95% CI)^a^		Ca/Co	OR (95% CI)^a^	
Oxygenated solvents exposure										
	Unexposed	99/176	1.00		196/302	1.00		53/40	1.00	0.42
	Exposed	30/40	1.17 (0.66–2.07)		38/78	0.75 (0.48–1.18)		9/9	0.67 (0.21–2.11)	
Chlorinated solvents exposure										
	Unexposed	125/207	1.00		288/415				1.00	0.91
	Exposed	4/9	0.85 (0.25–2.88)		8/14				0.99 (0.40–2.46)	
Fuels & petroleum-based solvents exposure										
	Unexposed	115/206	1.00		277/392				1.00	0.01
	Exposed	14/10	2.74 (1.11–6.76)		19/37				0.65 (0.35–1.18)	

^a^ Estimates obtained comparing TGCT cases to group A and group B controls combined and adjusted for sibship size, being born from multiple pregnancy, personal history of testicular trauma, family history of TGCT and family history of cryptorchidism. Analysis was restricted to subjects with no missing data for the adjustment variables (N=12). ^b^ If the categories of birth decades have less than 5 cases or controls, the categories “1981–90” and “1991–99” were combined into a single category (1981–99), otherwise the line is shaded.

The PCA approach identified 2–3 PC depending on the solvent and the parent. None of them showed an association with TGCT risk, confirming the absence of association between parental solvent exposure and TGCT risk in their son (supplementary tables S6 – S7).

In supplementary analyses, the results remained globally unchanged when we excluded cases with a personal history of cryptorchidism in the model (supplementary table S8) or when we excluded TGCT cases not confirmed by pathology reports (supplementary table S9). Further adjustment for age did not modify the results (supplementary table S10). The ORadj showed no substantial change from the crude OR (data not shown).

## Discussion

This study documents the relationship between parental occupational exposure to solvents and TGCT risk among their offspring in France. Parental occupation at birth was considered as a proxy of solvent exposure before and at conception (37). No solid evidence of an association between parental occupational exposure to solvents at birth and TGCT among sons was found. However, an increased risk with maternal exposure to petroleum-based solvents in the 1970s was observed. Our results may also suggest a modest increased risk of non-seminoma for sons whose fathers were highly exposed to TCE.

Prevalence of exposure to at least one oxygenated solvent reported in our study was higher among fathers but similar in mothers (ie, 24% fathers and 19% mothers) (supplementary table S5) than those observed in the population of French workers in 1999. (ie, 9% of men and 16% of women) (38). The prevalence of maternal exposures to specific solvents were lower than those reported for women during similar periods in another French case–control study (ICARE study) that also used the Matgéné JEM (39). The prevalence of paternal exposures were slightly higher than those assessed for men during similar periods for chlorinated solvents in the ICARE study (40), particularly for TCE (8% for controls in ICARE study), based on the whole career.

Some solvents, and other EDC, can cross the placental barrier, enter the fetal circulation (8), and interfere with hormone levels during fetal development (41). It has also been reported in two previous animal studies that fetal exposure to toluene and styrene was associated with reduced testosterone synthesis and secretion in the fetal testes and decreased male reproductive organ weights (42, 43). Nevertheless, in our study, no overall association between maternal occupational exposure to solvents at birth and TGCT in their offspring was observed. The exception was a positive association with maternal exposure to petroleum-based solvents in the 1970s. This finding must be interpreted with caution, as the number of mothers exposed was limited, and the CI was wide indicating substantial uncertainty around available estimates. However, this result was consistent with those observed in the two NORD-TEST studies (21, 22), particularly with the higher risk of TGCT (OR 1.44, 95% CI 1.00–2.08) for maternal exposure to aromatic hydrocarbon solvents (ie, petroleum solvents) among sons born in 1970–1979 shown in Denmark NORD-TEST study. Nevertheless, because of insufficient numbers, it was not possible to identify in our study whether this positive association observed for petroleum-based solvents in the 1970s was mainly driven by the aromatic hydrocarbon solvent category, as in NORD-TEST studies.

The association between maternal occupational solvents exposure and congenital malformations has been investigated in previous studies (11, 44–51). Contrasting results between maternal solvent exposures and hypospadias were observed (11, 45, 52). Overall, petroleum-based solvents have frequently been found to be associated with birth defects. Moreover, an experimental study in laboratory animals suggested that rats exposed prenatally to toluene have an increased risk of cryptorchidism (53). Thus, it seems possible that maternal exposure to solvents increases the risk of two other TDS conditions.

The role of paternal exposure to organic solvents has received less attention. Mechanisms by which male occupational exposure may cause reproductive and developmental defects are still largely unknown, however, it was suggested that transmission may also occur via epigenetic changes from father to child (19). The evidence for epigenetic alterations caused by carcinogens, and the potential association with genotoxic endpoints, is rapidly growing (19). Recently, exposure to TCE has been shown to cause changes in sperm histones in rats (18). These results raise the question of the role of epigenetic inheritance through histone modifications in the offspring after TCE exposure. Our findings also showed a modest positive association with non-seminoma and paternal TCE exposure, although CI were wide and included negative and null association values. The NORD-TEST study also observed a modest increase in the risk of TGCT in sons whose fathers were exposed to TCE, although the OR was higher in the low-TCE exposure category in this study (OR 1.12, 95%CI 0.96– 1.32). As is our study, no association between fathers not exposed/exposed or not exposed/low/highly exposed to other solvents was observed in the two previous Nordic registry-based studies (21, 22).

In France, the occupational exposure limit (OEL) of 8 hours (ie, average exposure limit value) in effect at the time of the relevant exposures, was set at 75 ppm for TCE (1983–2020), 50 ppm for PCE (until 2012). None of the exposure levels observed for fathers and mothers to these three solvents in our study were above the established OEL (supplementary table S6). Nevertheless, the OEL have decreased or regulated over time, and were set to date at 10 ppm for TCE (updated in 2021) at 20 ppm for PCE (2012) and at 50 ppm for MC (regulated since 2012) in France. About 5% of fathers in this study were exposed to TCE above the OEL. This could potentially explain the increase in TGCT risk suggested by our findings, albeit modest and with wide CI, for offspring whose fathers were highly exposed to TCE. Some mothers were also exposed to TCE (N=13), PCE (N=1) above the currently set OEL. Nevertheless, very few mothers were exposed to chlorinated solvents in our study, then it is possible that the power of the study did not allow us to show an increased risk of TGCT for sons whose mothers were exposed to these solvents.

Our study has several strengths. JEM applied in this study were developed by experienced industrial hygienists, specifically for the French population (28). Matgéné’s JEM integrate the probability of exposure, semi-quantitative indices of exposure level and the period of exposure, which allows the assignment of exposure to evolve over time, in connection with the evolution of techniques, occupation conditions and regulations. We were also able to adjust for a significant number of known or suspected risk factors, which had not been the case in previous studies of the association between parental exposures to solvents and TGCT risk in their offspring. However, information about other suspected risk factors, such as maternal bleeding during pregnancy or maternal smoking, was not available in our study (31). Therefore, it cannot be excluded that the associations reported in this study may be related to these other factors.

This study has also some limitations that may affect the interpretation of the results. In general, young men are known to be a difficult population to approach and less likely to participate in research than other population groups (54). Thus, low response rates make difficult ascertaining a population-based control group representative of the general population. Since no perfect control group was found, we choose two distinct control groups to test our hypotheses on populations presenting different aspects of the general population, as proposed by Stang et al (55). Hospital-based control recruitment aims to facilitate the recruitment of our young male population, as well as the management of biological sampling. Moreover, according to the TDS hypothesis, TGCT, cryptorchidism, hypospadias and poor semen quality correspond to the same prenatal alteration but with a different clinical expression (9). By choosing controls supposed to be fecund [Group B (partners of pregnant woman hospitalized for pathological pregnancy) recruited at the level III maternity hospital] or having a normal sperm count [Group A (sperm donors & fertile partners of infertile woman) recruited at CECOS], we reduced the risk of having controls with minor forms of this syndrome. Yet, consequently, we cannot exclude that our controls may be more fertile than the general population of the same age (54). Data is limited in defining if this has biased the results. However, to affect the associations examined in the present study, it would require that factors responsible for increased fertility in the controls are non-occupational albeit correlated with parental occupations. Although prospective recruitment of cases from one of the participating CECOS for sperm cryopreservation appears to be the most appropriate method to ensure good response rate in the present study (54), this recruitment has some limitations. Cases who presented for sperm cryopreservation prior to cancer treatment may not be representative of all men with TGCT, such as those who are already fathers, or those with azoospermia may be underrepresented. Moreover, occupational exposures were assessed by JEM, which provide an average assessment for an ISCO-NAF pair by considering the variability of exposures within the same job. However, within the same ISCO-NAF pair, there may be jobs with varying intensity and frequency of exposure. A limitation of JEM is that they do not account for individual variation on exposure within the same job, which may generate non-differential misclassification of exposure compared to an individual assessment, and could result in bias toward the null (56). It is important to note that our results may be limited in terms of statistical power because of the low prevalence of some exposure variables and small numbers in the subgroup analysis, leading to effect estimates with high statistical uncertainty. Despite the consistency of the primary and secondary analyses, we cannot rule out the possibility that our results are due to chance because of multiple testing. It should be noted that the few associations identified in this study presented plausible hypotheses consistent with the results of previous experimental and human studies (18, 21, 22). Finally, because of the study design, we had to assume that the parental job at birth reflected the exposure at the time of conception and during intrauterine development, following the example of other studies (21, 22, 37) even though a change in employment may have occurred during these periods. In the TESTIS study, upon written consent by the participants, the participants’ mothers were also contacted to offer them participation in the study and a telephone interview. In this interview, like the participants, the mothers described their work history as well as that of the father. As a measure of quality, agreement between parental employments at birth provided by the participants and the ones provided by the mothers was estimated, using Cohen’s kappa coefficient (57). For this analysis, data from 547 mothers (50% participation rate in the two-stage recruitment process of case/control mothers/relatives; 75% participation rate of mothers for which the participating sons agreed to provide the contact) (24) were used. Kappa values ranged from moderate agreement for the more specific coding levels to perfect agreement for the more general coding levels. The primary analysis was conducted with subjects’ reported birth parental occupations (son) to maximize the sample size and therefore the power of the study.

### Concluding remarks

Overall, this study suggests no substantial role of parental occupational exposure to solvents and TGCT risk, although maternal occupational exposure to petroleum-based solvents suggested a positive association among men born in the 1970s. Paternal occupation to high levels of TCE showed a slightly elevated non-seminoma risk that needs to be confirmed by further studies, which also requires research on the molecular mechanisms involved.

### Collaborators

Rémi Béranger, Helen Boyle, Aude Fléchon, and Elodie Belladame; Céline Chalas, Vanessa Gayet, Paul Pirtea, Pietro Santulli, Aurélie Vincent, Edouard Lecarpentier, François Goffinet, Dominique De Ziegler, Khaled Pocate, Virginie Barraud-Lange, Jean-Philippe Wolf, Emmanuel Dulioust, Nathalie Le Foll, Jacques Auger, Anne-Sophie Gille, Laurianne Kremer, Myriam Virlouvet, Lucile Ferreux, Guillemette Perier, Pauline Peretout, Diane Rivet, Véronique Drouineaud and Sandrine Rulle (Cochin Hospital, Paris); Rachel Levy, Nathalie Sermondade, Yassine Belaid, Marine Durand and Charlène Harbemont (Jean Verdier Hospital, Paris); Xavier Pollet-Villard, Vanina De Larouziere, Laurence Levy-Dutel, Florence Eustache and Isabelle Berthaut (Tenon Hospital, Paris); Jacqueline Saias-Magnan, Jeanne Perrin, Catherine Metzler-Guillemain, Carole Daoud-Deveze and Laurent Nasca (La Conception Hospital, Marseille); Myriam Daudin, Nathalie Moinard, François Isus, Célia Bettiol and Laure Connan (Paule de Viguier Hospital, Toulouse); Laurent Janny, Valérie Bruhat, Florence Brugnon and Cyril Bouche (Estaing Hospital, Clermont-Ferrand); Laëtitia Ladureau-Fritsch, Cécile Greze, Françoise Schmitt, and Charles Pax (Obstetric medico-surgical center, Strasbourg); Clément Jimenez, Volcy Soula, Lucie Chansel and Olivier Delorme (Pellegrin maternity Hospital, Bordeaux); Sandrine Giscard d’Estaing, Pascale Dehee and Delphine Yalcinkaya (Femme-Mère-Enfant Hospital, Lyon); Céline Bouillon, Fabrice Guerif, Cynthia Frapsauce, Marie-Laure Couet, Véronique Ract, Olivia Gervereau, Elodie Poisson, Michel Lanoue, Anne Viallon and Catherine Guerin (Bretonneau Hospital, Tour); Bérengère Ducrocq, Julie Guitton, Marie Lefort and Valérie Mitchell (Calmette Hospital, Lille); Marie-Ange Clarotti, Ethel Szerman, Amélie Ancelle, Catherine Muris, Corinne Fourmy-Chatel, Christine Denoual-Ziad, Claire De Vienne, Cécile Delesalle, Jean-Paul Bouiller and Antoine Clergeau (Caen Hospital, Caen); Alphée Bailly and Séverine Bey (Jean Minjoz Hospital, Besançon); Célia Ravel, Guilhem Jouve, Ségolène Veau, Laurent Vandenbroucke and Agnès Letremy (South Hospital, Rennes); Julie Barberet (Dijon Hospital, Dijon); Stéphanie Lattes, Emmanuelle Thibault, Fabienne Bernardin, Pierre Besnier and Clémence Martin (Archet Hospital, Nice); Catherine Diligent, Françoise Touati, Nicolas Monnin and Christel Hersant (Regional University maternity Hospital, Nancy); Vanessa Loup-Cabaniols, Alice Ferrieres, Anna Gala, Elodie Scalici, Lucile Sablayrolles, Tiffany Mullet, Audrey Chabert, Christelle Saintpeyre, Mélanie Caro, Michèle Nou and Marie Sicard (Arnaud de Villeneuve Hospital, Montpellier); Sylvianne Hennebicq, Pascale Hoffmann, Claire Thomas-Cadi, Nicole Quenard, Evelyne Warembourg, Laure Villaret and Julien Bessonnat (Couple-Enfant Hospital, Grenoble); Marie-Claude Blocquaux, Frédérique Carre-Pigeon, Béatrice Delepine, Olivier Graesslin and Julie Burette (Maison Blanche Hospital, Reims).

### Contributors

AD, OP, LB, JS, BF and BC participated in the conception and design of the study. AD, OP, JS, LB, IK, AP, OB, PF, BF and BC participated in the acquisition of data. MG, ML, CP, AC, SA, AD, BD, AO, BF and BC participated in the analysis and interpretation of data. MG drafted the manuscript. ML, CP, AC, AD, AO, JS, BF and BC critically revised the manuscript for important intellectual content. All authors read and approved the final manuscript.

### Disclaimer

Where authors are identified as personnel of the International Agency for Research on Cancer/World Health Organization (IARC/WHO), they alone are responsible for the views expressed in this article, which do not necessarily represent the decisions, policy or views of the IARC/WHO.

### Ethics approval

The study received ethical approval from the French Ethics Committee (ref. no. A14–94), the French national agency for medicines and health products safety (ref. no. 140184B-12) and the IARC Ethics Committee (ref. no. 14–26) and was declared to the Commission nationale Informatique et Libertés (MR-001, ref. no. 2016–177).

## Supplementary material

Supplementary material
