# Supplementary material for: Parental occupational exposure to solvents and risk of developing testicular germ cell tumors among sons: a French nationwide case-control study (TESTIS study)
Source: Scand J Work Environ Health. 2023 Aug 29;49(6):405–18. doi: 10.5271/sjweh.4102 (PMC10812531; doi:10.5271/sjweh.4102)
Supplement: Supplementary material [file SJWEH-49-405-S001.pdf]

Parental occupational exposure to solvents and risk of developing testicular germ cell tumors among sons: a French nationwide case-control study (TESTIS study)[1]

by Margot Guth, MSc, Marie Lefevre, MSc, Corinne Pilorget, PhD, Astrid Coste, PhD, Shukrullah Ahmadi, PhD, Aurélie Danjou, PhD, Brigitte Dananché, DipEng, Delphine Praud, PhD, Isabelle Koscinski, PhD, Aline Papaxanthos, PhD, Oxana Blagosklonov, PhD, Patricia Fauque, PhD, Olivia Pérol, Msc, Joachim Schüz, PhD, Louis Bujan, PhD,Ann Olsson, PhD, Béatrice Fervers, PhD,[2] Barbara Charbotel, PhD and the TESTIS study group

1. *Supplementary material*
2. *Corresponce to: Béatrice Fervers - Prevention Cancer Environnement Departement, Centre Léon Bérard, Lyon, France. [E-mail: beatrice.fervers@lyon.unicancer.fr]*

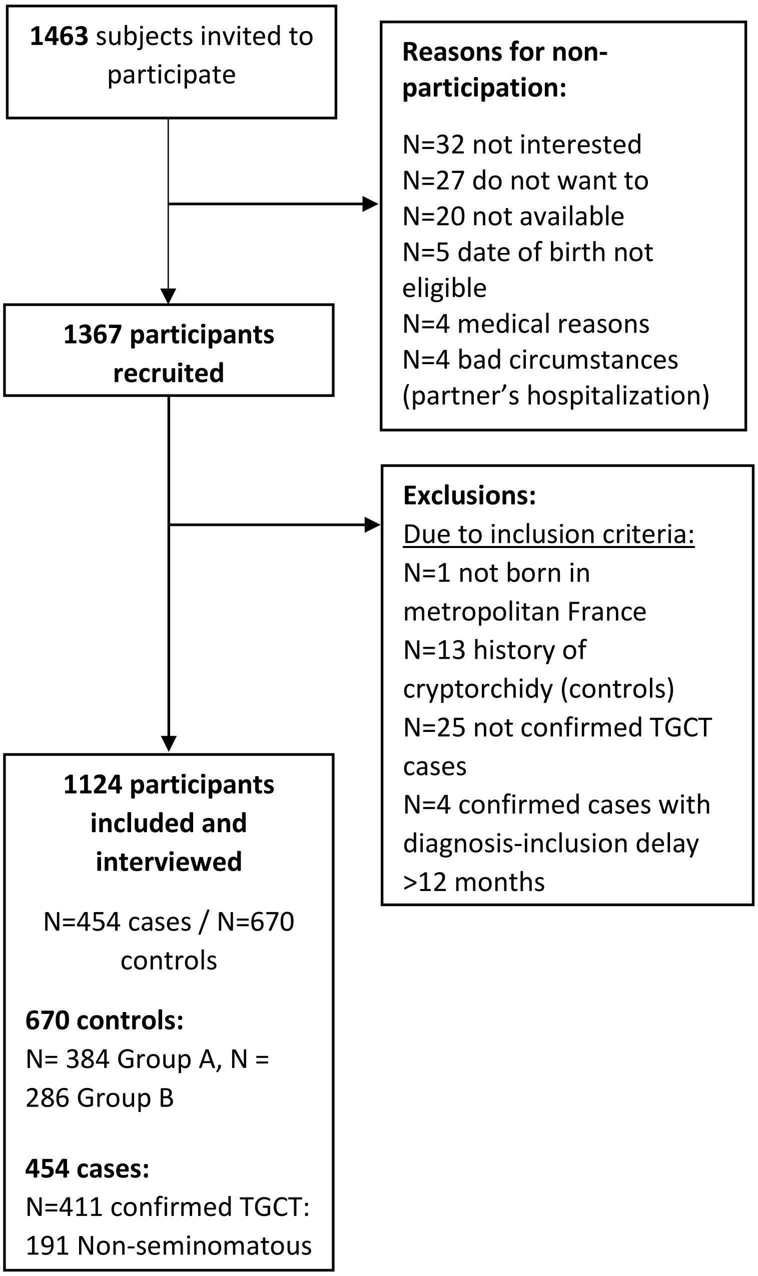

Figure S1. Flow chart of the TESTIS study population

Table S1. Indices from JEMs for all solvents and weight values used for the parental exposure estimates calculations.

Probability of exposure	Weight [a]	Frequency of exposure	Weight [a]	Solvent	Intensity of exposure	Weight [a]	At least one solvent: Level of exposure	At least one solvent: Weight [a]
**Chlorinated solvents**								
< 1%	**0**	< 1%	**0**		< 5 ppm	**0**	not exposed	**0**
[1 - 10%[	**0.05**	[1 - 10%[	**0.05**		[5 – 25 ppm]	**15**	very low	**15**
[10 - 20%[	**0.15**	[10 - 20%[	**0.15**	**Trichloroethylene, Perchloroethylene, Methylene chloride**	[26 – 50 ppm]	**37.5**	low	**37.5**
[20 - 30%[	**0.25**	[20 - 30%[	**0.25**		[51 – 100 ppm]	**75**	medium	**75**
[30 - 39%[	**0.35**	[30 - 40%[	**0.35**		> 100 ppm	**150**	high	**150**
[40 – 50%[	**0.45**	[40 – 50%[	**0.45**		not exposed	**0**		
[50 - 60%[	**0.55**	[50 - 60%[	**0.55**		very low	**0.625**		
[60 - 70%[	**0.65**	[60 - 70%[	**0.65**	**Chloroform**	low	**1.875**		
[70 - 80%[	**0.75**	[70 - 80%[	**0.75**		medium	**3.75**		
[80 - 90%[	**0.85**	[80 – 90%[	**0.85**		high	**10**		
≥ 90%	**0.95**	≥ 90%	**0.95**		not exposed	**0**		
					very low	**0.25**		
				**Carbon tetrachloride**	low	**0.75**		
					medium	**1.5**		
					high	**3**		
**Fuels and Petroleum-based solvents**								
< 1%	**0**	< 0.5%	**0**		0	**0**	not exposed	**0**
[1 - 10%[	**0.05**	[0.5 – 5%[	**0.025**		[0.1 – 1 ppm]	**0.5**	low	**3.5**
[10 - 50%[	**0.30**	[5 – 30%[	**0.175**	**Benzene**	]1 – 5 ppm]	**3**	medium	**23**
[50 - 90%[	**0.70**	[30 – 70%[	**0.50**		]5 – 15 ppm]	**10**	high	**70**
[90 - 100%]	**0.95**	≥ 70%	**0.85**		> 15 ppm	**20**		
					< 1 ppm	**0**		
					[1 – 20 ppm]	**10**		
				**White spirits and other aromatics**	]20 – 50 ppm]	**35**		
					> 50 ppm	**65**		
					not exposed	**0**		
				**Special petroleum products and other aliphatic**	low	**10**		
					medium	**35**		
					high	**65**		
					< 1 ppm	**0**		
					[1 – 50 ppm]	**25**		
				**Gasoline**	]50 – 150 ppm]	**100**		
					> 150 ppm	**200**		
					not exposed	**0**		
				**Kerosene/diesel oil/fuel oils**	low	**25**		
					medium	**100**		
					high	**200**		
**Oxygenated solvents**								
< 1%	**0**	< 1%	**0**		not exposed	**0**	not exposed	**0**
1 - 9%	**0.05**	1 - 9%	**0.05**		very low	**1**	very low	**1**
10 - 19%	**0.15**	10 - 19%	**0.15**	**Alcohol, Ketones/esters**	low	**4**	low	**4**
20 - 29%	**0.25**	20 - 29%	**0.25**		medium	**8**	medium	**8**
30 - 39%	**0.35**	30 - 39%	**0.35**		high	**16**	high	**16**
40 - 49%	**0.45**	40 - 49%	**0.45**		not exposed	**0**		
50 - 59%	**0.55**	50 - 59%	**0.55**		low	**1**		
60- 69%	**0.65**	60 - 69%	**0.65**	**Diethyl ether, Ethylene glycol, Tetrahydrofuran**	medium	**3**		
70 - 79%	**0.75**	70 - 79%	**0.75**		high	**6**		
80 - 89%	**0.85**	80 - 89%	**0.85**					
≥ 90%	**0.95**	≥ 90%	**0.95**					

[a] Weights have been used to calculate parental exposure estimates (PEEs).

Table S2. The cut-offs of paternal and maternal solvent exposure calculated from parental exposure score, TESTIS study, 2015-2018.

	50th percentiles values of mothers' exposures [a]	50th percentiles values of fathers' exposures [a]
**Oxygenated solvents**	0.95	0.58
Alcohols	0.57	0.34
Ketones and esters	0.25	0.21
Ethylene glycol	0.15	0.29
Diethylether	0.02	0.02
Tetrahydrofurane	0.01	0.01
**Chlorinated solvents**	3.75	2.25
Trichloroethylene	0.84	0.84
Perchloroethylene	0.38	0.52
Methylene chloride	0.94	1.31
Chloroform	0.005	0.01
Carbon tetrachloride	0.002	0.002
**Fuels & Petroleum-based solvents**	0.18	1.05
Benzene	0.003	0.03
Automobile gasoline	1.25	0.44
Special petroleum products	0.02	0.08
White spirits	0.01	0.08
Diesel, kerosene, fuel oil	0.05	0.44

[a] Mothers' exposures and fathers' exposure were calculated as the product of probability of exposure to the solvent (P), intensity of exposure (I) and frequency of exposure (F), using the central value of the classes (weight Table S1). For exposure to at least one solvent within the same family, average level (L) was used in calculation (product of P and L).

Table S3. Most held occupations in parents, by exposure estimates to oxygenated, chlorinated and fuel and petroleum-based solvents, TESTIS study, 2015-2018.

Parental exposure estimates [c]	Fathers (N=1077)		Mothers (N=1088)	
	N [a]	Two main jobs for each ISCO68 main group (% of jobs) [b]	N [a]	Two main jobs for each ISCO68 main ISCO68 (% of jobs) [b]
**Oxygenated solvents**				
0	**815**	Technical Salesman (3.7), Lorry and Van Driver (Local Transport) (2.9)	**1053**	Office Clerk (6.4), Stenographer-Typist (4.8)
1	**207**	Specialized Physician (6.8), General Physician (5.8)	**22**	Retail Trade Salesman (22.7), Machine Packer (9.1)
2	**55**	Construction Joiner (21.8), Printer (General) (9.1)	**13**	Electrical Equipment Assembler (23.1), Biologist (15.4)
**Chlorinated solvents**				
0	**885**	Technical Salesman (3.4), Lorry and Van Driver (Local Transport) (2.7)	**878**	Office Clerk (7.6), Stenographer-Typist (5.8)
1	**148**	Automobile Mechanic (8.1), Construction Joiner (6.8)	**191**	Nursing Aid (17.8), Charworker (16.8)
2	**44**	Plant Maintenance Mechanic (13.6), Electronics Fitter (Industrial Equipment) (11.4)	**19**	Women's Hairdresser (15.8), Biologist (10.5)
**Fuels & Petroleum-based solvents**				
0	**765**	Technical Salesman (3.9), General Farmer (3.0)	**1008**	Office Clerk (6.7), Stenographer-Typist (5.1)
1	**200**	Construction Joiner (6.0), Motor-Vehicle Drivers (6.0)	**60**	Charworker (53.3), Retail Trade Salesman (8.3)
2	**49**	Lorry and Van Driver: local (44.9), long distance (30.6)	**20**	Medical Science Technician (10.0), Life Sciences Technicians (10.0)

[a] Total number of jobs in that category.

[b] 2 main job categories according to ISCO-68 classification and percentage of the total number of jobs in in each parent exposure estimates class.

[c] Parental exposure estimates were calculated as the product of the proportion of exposed workers (P) within job and the level of exposure to the agent among the exposed workers (L); 0: “Not exposed”, 1: “Low exposed”, 2: “High exposed”.

Table S4. Correlation matrix between different solvents for mothers (white cells) and fathers (grey cells) occupationally exposed, TESTIS study, 2015-2018.

	Alc	Ket Est	THF	Glycol	Et2O	MC	CF	PCE	CT	TCE	GL	SBPs	BZ	KDF	WS
Alc	**1.00**	0.49	0.18	0.03	0.29	0.35	0.20	0.05	0.13	0.21	0.08	0.25	0.25	0.00	0.28
Ket Est	0.31	**1.00**	0.41	0.47	0.13	0.51	0.22	0.10	0.16	0.49	0.39	0.33	0.55	0.19	0.54
THF	0.08	0.15	**1.00**	-0.02	0.05	0.06	0.07	-0.02	0.10	0.21	-0.03	0.06	0.04	-0.05	0.26
Glycol	0.07	0.26	0.00	**1.00**	0.03	0.08	0.05	-0.02	-0.01	0.31	0.59	0.01	0.51	0.41	0.28
Et2O	0.69	0.07	0.10	0.04	**1.00**	0.31	0.62	-0.02	0.47	-0.03	0.00	0.22	0.17	-0.02	0.03
MC	0.26	0.55	0.26	0.14	0.18	**1.00**	0.51	0.21	0.38	0.29	0.13	0.35	0.40	0.06	0.32
CF	0.22	0.43	0.31	0.18	0.24	0.81	**1.00**	-0.01	0.74	-0.01	0.02	0.41	0.32	0.00	0.11
PCE	0.08	0.23	0.00	0.00	-0.02	0.25	-0.01	**1.00**	-0.01	0.34	-0.04	0.14	0.10	-0.05	0.24
CT	0.18	0.40	0.38	0.00	0.26	0.68	0.83	0.00	**1.00**	-0.03	-0.02	0.31	0.24	-0.03	0.08
TCE	0.06	0.55	0.00	0.22	-0.03	0.30	0.05	0.30	-0.01	**1.00**	0.28	0.24	0.35	0.14	0.70
GL	0.07	0.26	0.00	1.00	0.04	0.14	0.18	0.00	0.00	0.22	**1.00**	0.14	0.56	0.51	0.30
SBPs	0.16	0.46	0.30	0.17	0.16	0.46	0.57	0.15	0.56	0.29	0.17	**1.00**	0.52	0.02	0.33
BZ	0.18	0.48	0.31	0.55	0.17	0.56	0.59	0.15	0.59	0.24	0.55	0.66	**1.00**	0.30	0.42
KDF	-0.01	0.31	0.00	0.45	-0.01	0.05	0.07	-0.01	-0.01	0.35	0.45	0.07	0.24	**1.00**	0.22
WS	0.38	0.30	0.12	0.14	0.00	0.34	0.23	0.18	0.23	0.35	0.14	0.33	0.35	0.11	**1.00**

Abbreviations – Alc: Alcohols; KetEst: Ketones and esters; THF: Tetrahydrofurane; Glycol: Ethylene glycol; Et2O: Diethylether; MC: Methylene chloride; CF: Chloroform; PCE: Perchloroethylene ; CT: Carbon tetrachloride ; TCE: Trichloroethylene; GL: gasoline ; SBPs: special petroleum products and other aliphatics; BZ: Benzene ; KDF: kerosene/diesel oil/fuel oils; WS: White spirits.
Oxygenated solvents are shown in green, chlorinated solvents in orange and fuels & petroleum-based solvents in blue.

Table S5. Prevalence of parental occupational exposure to organic solvents for cases and controls, TESTIS study, 2015-2018.

Solvents	**Fathers**								**Mothers**							
	Cases (N=413) exposed		Group A controls (N=370) exposed		Group B controls (n=271) exposed		Subjects (N=1054) exposed		Cases (N=425) exposed		Group A controls (N=368) exposed		Group B controls (N=277) exposed		Subjects (N=1070) exposed	
	N	%	N	%	N	%	N	%	N	%	N	%	N	%	N	%
**Any solvents**	162	39.23	169	45.7	95	35.1	426	40.42	84	19.76	80	21.7	56	20.2	220	20.56
**Oxygenated solvents**	87	21.07	102	27.6	66	24.4	255	24.19	77	18.12	72	19.6	55	19.9	204	19.07
Alcohols	52	12.59	64	17.3	38	14.0	154	14.61	73	17.18	69	18.8	52	18.8	194	18.13
Ketones and esters	32	7.75	41	11.1	23	8.5	96	9.11	15	3.53	14	3.8	12	4.3	41	3.83
Diethylether									33	7.76	30	8.2	27	9.7	90	8.41
Ethylene glycol	9	2.18	11	3.0	6	2.2	26	2.47								
**Chlorinated solvents**	69	16.71	76	20.5	46	17.0	191	18.12	12	2.82	13	3.5	10	3.6	35	3.27
Trichloroethylene	67	16.22	73	19.7	43	15.9	183	17.36	8	1.88	10	2.7	7	2.5	25	2.34
Perchloroethylene	10	2.42	8	2.2	2	0.7	20	1.90								
Methylene chloride	14	3.39	12	3.2	7	2.6	33	3.13								
**Fuels & Petroleum-based solvents**	118	28.57	121	32.7	62	22.9	301	28.56	33	7.76	30	8.2	17	6.1	80	7.48
Benzene	17	4.12	22	5.9	9	3.3	48	4.55								
Automobile gasoline	24	5.81	30	8.1	13	4.8	67	6.36								
Special petroleum products	13	3.15	13	3.5	5	1.8	31	2.94								
White spirits	77	18.64	91	24.6	50	18.4	218	20.68	26	6.12	26	7.1	15	5.4	67	6.26
Diesel, kerosene, fuel oil	58	14.04	47	12.7	29	10.7	134	12.71	7	1.65	5	1.4	3	1.1	15	1.40

When the number of subjects was considered insufficient, i.e., less than 5 cases or 5 controls, the prevalence was not calculated (shaded line).

Table S6. Odds ratios and 95% confidence intervals for TGCT associated with paternal occupational exposure using solvent principal component analysis, TESTIS study, 2015-2018.

	All TGCT cases			Non-seminomas			Seminomas			Crude p-het	*Adjusted p-het*
	Ca/Co	Crude OR (95% CI) [a]	*Adjusted OR (95% CI)* [b]	Ca/Co	Crude OR (95% CI) [a]	*Adjusted OR (95% CI)* [b]	Ca/Co	Crude OR (95% CI) [a]	*Adjusted OR (95% CI)* [b]		
**Oxygenated solvents**	413/641			170/641			201/641				
Component 1: composed by Alcohols, Ketones and esters		1.00 (0.90-1.10)	1.00 (0.91-1.11)		1.01 (0.88-1.17)	1.01 (0.87-1.17)		0.98 (0.87-1.10)	0.98 (0.87-1.10)	0.73	0.77
Component 2: composed by Ethylene glycol		0.99 (0.87-1.12)	0.97 (0.85-1.11)		1.01 (0.85-1.19)	1.01 (0.85-1.19)		0.92 (0.75-1.14)	0.90 (0.72-1.12)	0.53	0.43
Component 3: Diethylether, Tetrahydrofurane		1.01 (0.89-1.14)	1.01 (0.88-1.15)		1.07 (0.93-1.23)	1.08 (0.93-1.24)		0.97 (0.78-1.20)	0.98 (0.78-1.23)	0.46	0.48
**Chorinated solvents**	413/641			170/641			201/641				
Component 1: Trichloroethylene, Perchloroethylene		1.01 (0.92-1.10)	0.97 (0.85-1.11)		1.03 (0.91-1.17)	1.01 (0.89-1.15)		0.98 (0.87-1.11)	0.94 (0.83-1.07)	0.55	0.44
Component 2: Carbon tetrachloride, Chloroform, Methylene chloride		1.01 (0.92-1.12)	1.01 (0.88-1.15)		0.93 (0.75-1.16)	0.93 (0.75-1.15)		0.99 (0.85-1.14)	0.99 (0.86-1.14)	0.66	0.63
**Fuels & petroleum-based solvents**	413/641			170/641			201/641				
Component 1: composed by Benzene, Diesel/Kerosene/Fuel oil, Automobile gasoline		1.00 (0.92-1.08)	0.98 (0.90-1.07)		0.99 (0.89-1.11)	1.00 (0.89-1.12)		0.94 (0.82-1.08)	0.91 (0.79-1.06)	0.53	0.33
Component 2: composed by Special petroleum products, White spirits		1.00 (0.90-1.13)	1.01 (0.90-1.14)		1.00 (0.84-1.20)	1.00 (0.84-1.20)		1.03 (0.91-1.17)	1.04 (0.91-1.19)	0.80	0.73

Abbreviations - OR: odds ratio; 95% CI: confidence interval of 95%; Ca/Co: Cases/controls; p-het: p for heterogeneity.

[a] Estimates obtained comparing TGCT cases to group A and group B controls combined.

[b] Estimates obtained comparing TGCT cases to group A and group B controls combined and adjusted for sibship size, being born from multiple pregnancy, personal history of testicular trauma, family history of TGCT and family history of cryptorchidism.

Table S7. Odds ratios and 95% confidence intervals for TGCT associated with maternal occupational using solvent principal component analysis, TESTIS study, 2015-2018.

	All TGCT cases			Non-seminomas			Seminomas			Crude p-het	*Adjusted p-het*
	Ca/Co	Crude OR (95% CI) [a]	*Adjusted OR (95% CI)* [b]	Ca/Co	Crude OR (95% CI) [a]	*Adjusted OR (95% CI)* [b]	Ca/Co	Crude OR (95% CI) [a]	*Adjusted OR (95% CI)* [b]		
**Oxygenated solvents**	425/645			173/645			212/645				
Component 1: composed by Alcohols, Ketones and esters		1.00 (0.91-1.10)	1.01 (0.92-1.12)		0.96 (0.81-1.14)	0.95 (0.80-1.13)		1.02 (0.91-1.14)	1.03 (0.92-1.15)	0.56	0.47
Component 2: composed by Diethylether, Ethylene glycol and Ketones and esters		1.02 (0.90-1.15)	1.02 (0.89-1.16)		0.99 (0.82-1.19)	0.99 (0.82-1.20)		1.01 (0.88-1.16)	0.99 (0.86-1.15)	0.84	0.98
**Chorinated solvents**	425/645			173/645			212/645				
Component 1: Trichloroethylene, Perchloroethylene		1.01 (0.92-1.10)	1.01 (0.92-1.11)		1.04 (0.92-1.17)	1.03 (0.91-1.17)		0.99 (0.88-1.11)	0.99 (0.88-1.11)	0.56	0.64
Component 2: Carbon tetrachloride, Chloroform, Methylene chloride		1.01 (0.91-1.12)	1.02 (0.91-1.13)		1.05 (0.92-1.19)	1.06 (0.92-1.21)		0.95 (0.81-1.12)	0.96 (0.81-1.13)	0.36	0.36
**Fuels & petroleum-based solvents**	425/645			173/645			212/645				
Component 1: composed by Benzene, Diesel/Kerosene/Fuel oil, Automobile gasoline		1.00 (0.93-1.07)	0.99 (0.92-1.06)		0.95 (0.76-1.18)	0.94 (0.74-1.20)		1.01 (0.94-1.08)	0.99 (0.91-1.07)	0.49	0.64
Component 2: composed by Special petroleum products, White spirits		1.01 (0.91-1.13)	1.02 (0.92-1.14)		1.07 (0.95-1.21)	1.06 (0.94-1.21)		0.94 (0.79-1.12)	0.96 (0.82-1.14)	0.33	0.22

Abbreviations - OR: odds ratio; 95% CI: confidence interval of 95%; Ca/Co: Cases/controls; p-het: p for heterogeneity.

[a] Estimates obtained comparing TGCT cases to group A and group B controls combined.

[b] Estimates obtained comparing TGCT cases to group A and group B controls combined and adjusted for sibship size, being born from multiple pregnancy, personal history of testicular trauma, family history of TGCT and family history of cryptorchidism.

Table S8. Odds ratios and 95% confidence intervals for TGCT associated with parental occupational exposure to solvents in the year of the child's birth, when excluding cases with personal history of cryptorchidism, TESTIS study, 2015-2018.

Solvents exposure		Paternal exposure			Maternal exposure		
		Ca/Co	Crude OR (95% CI) [a]	*Adjusted OR (95% CI)* [b]	Ca/Co	Crude OR (95% CI) [a]	*Adjusted OR (95% CI)* [b]
**Oxygenated solvents**	None	297/473	1.00	1.00	318/518	1.00	1.00
	Low	40/84	0.79 (0.52-1.20)	0.73 (0.48-1.12)	64/113	0.86 (0.61-1.22)	0.85 (0.60-1.21)
	High	37/84	0.69 (0.46-1.06)	0.70 (0.46-1.06)	5/14	0.67 (0.24-1.88)	0.73 (0.26-2.09)
Alcohols	None	330/539	1.00	1.00	320/524	1.00	1.00
	Low	20/51	0.67 (0.39-1.15)	0.63 (0.36-1.10)	67/121	0.85 (0.61-1.20)	0.85 (0.60-1.20)
	High	24/51	0.78 (0.47-1.30)	0.83 (0.50-1.40)			
Ketones and esters	None	343/577	1.00	1.00	374/619	1.00	1.00
	Low	19/44	0.78 (0.44-1.36)	0.76 (0.43-1.34)	13/13	0.91 (0.46-1.80)	0.91 (0.45-1.84)
	High	12/20	1.05 (0.50-2.22)	1.05 (0.49-2.23)			
Ethylene glycol	None	365/624	1.00	1.00			
	Low	9/17	0.84 (0.36-1.93)	0.75 (0.32-1.75)			
	High						
Diethylether	None				355/588	1.00	1.00
	Low				23/35	1.12 (0.64-1.97)	1.14 (0.65-2.01)
	High				9/22	0.68 (0.30-1.56)	0.68 (0.29-1.58)
**Chlorinated solvents**	None	309/519	1.00	1.00	377/622	1.00	1.00
	Low	29/66	0.70 (0.44-1.12)	0.66 (0.41-1.06)	10/23	0.76 (0.36-1.63)	0.78 (0.36-1.67)
	High	36/56	1.05 (0.67-1.65)	1.00 (0.63-1.58)			
Trichloroethylene	None	311/525	1.00	1.00	381/628	1.00	1.00
	Low	28/63	0.70 (0.43-1.13)	0.66 (0.40-1.07)	6/17	0.60 (0.23-1.55)	0.61 (0.23-1.58)
	High	35/53	1.07 (0.68-1.70)	1.03 (0.64-1.64)			
Perchloroethylene	None	365/631	1.00	1.00			
	Low	9/10	1.63 (0.64-4.11)	1.38 (0.52-3.67)			
	High						
Methylene chloride	None	360/622	1.00	1.00			
	Low	6/10	1.02 (0.36-2.89)	0.94 (0.33-2.72)			
	High	8/9	1.58 (0.59-4.19)	1.60 (0.59-4.31)			

Table S8 *continued*. Odds ratios and 95% confidence intervals for TGCT associated with parental occupational exposure to solvents in the year of the child's birth, when excluding cases with personal history of cryptorchidism, TESTIS study, 2015-2018.

Solvents exposure		Paternal exposure			Maternal exposure		
		Ca/Co	Crude OR (95% CI) [a]	*Adjusted OR (95% CI)* [b]	Ca/Co	Crude OR (95% CI) [a]	*Adjusted OR (95% CI)* [b]
**Fuels & Petroleum-based solvents**	None	269/458	1.00	1.00	357/598	1.00	1.00
	Low	64/105	1.04 (0.73-1.49)	1.04 (0.73-1.49)	22/35	0.93 (0.53-1.64)	0.90 (0.51-1.60)
	High	41/78	0.86 (0.57-1.31)	0.80 (0.53-1.23)	8/12	1.13 (0.45-2.82)	1.07 (0.42-2.)
Benzene	None	357/610	1.00	1.00			
	Low	7/16	0.89 (0.35-2.24)	0.77 (0.30-2.00)			
	High	10/15	1.17 (0.52-2.68)	1.06 (0.46-2.46)			
Automobile gasoline	None	350/598	1.00	1.00			
	Low	14/22	0.89 (0.43-1.80)	0.90 (0.44-1.84)			
	High	10/21	0.83 (0.38-1.81)	0.70 (0.31-1.57)			
Special petroleum products	None	361/623	1.00	1.00			
	Low<br>High	13/18	1.26 (0.60-2.65)	1.13 (0.53-2.40)			
White spirits	None	304/500	1.00	1.00	363/604	1.00	1.00
	Low	43/80	0.90 (0.60-1.34)	0.88 (0.58-1.32)	18/29	0.85 (0.46-1.58)	0.81 (0.43-1.51)
	High	27/61	0.74 (0.46-1.21)	0.75 (0.46-1.23)	6/12	0.88 (0.32-2.40)	0.93 (0.34-2.55)
Diesel, kerosene, fuel oil	None	322/565	1.00	1.00	381/637	1.00	1.00
	Low	41/50	1.46 (0.94-2.29)	1.52 (0.96-2.39)	6/8	1.43 (0.48-4.23)	1.30 (0.43-3.98)
	High	11/26	0.72 (0.34-1.51)	0.60 (0.28-1.29)			

Abbreviations - OR: odds ratio; 95% CI: confidence interval of 95%; Ca/Co: Cases/controls.

[a] Estimates obtained comparing TGCT cases to group A and group B controls combined.

[b] Estimates obtained comparing TGCT cases to group A and group B controls combined and adjusted for sibship size, being born from multiple pregnancy, personal history of testicular trauma, family history of TGCT and family history of cryptorchidism. Analysis was restricted to subjects with no missing data for the adjustment variables (N=12) and excluding cases with personal history of cryptorchidism (N=40). If the "Low"/"High" categories have less than 5 cases or controls, the categories are grouped into "None" or "All" if the number of subjects was sufficient, otherwise the line is shaded.

Table S9. Odds ratios and 95% confidence intervals for TGCT associated with parental occupational exposure to solvents in the year of the child's birth, when excluding cases not confirmed by pathology reports, TESTIS study, 2015-2018.

Solvents exposure		Paternal exposure			Maternal exposure		
		Ca/Co	Crude OR (95% CI) [a]	*Adjusted OR (95% CI)* [a,b]	Ca/Co	Crude OR (95% CI) [a]	*Adjusted OR (95% CI)* [a,b]
**Oxygenated solvents**	None	295/473	1.00	1.00	317/518	1.00	1.00
	Low	41/84	0.79 (0.53-1.19)	0.73 (0.48-1.12)	69/127	0.86 (0.62-1.20)	0.84 (0.60-1.18)
	High	36/84	0.67 (0.44-1.02)	0.70 (0.46-1.06)			
Alcohols	None	323/539	1.00	1.00	321/524	1.00	1.00
	Low	23/51	0.76 (0.45-1.28)	0.63 (0.36-1.10)	65/121	0.84 (0.60-1.19)	0.81 (0.57-1.16)
	High	26/51	0.83 (0.51-1.38)	0.83 (0.50-1.40)			
Ketones and esters	None	344/577	1.00	1.00	373/619	1.00	1.00
	Low	17/44	0.70 (0.39-1.25)	0.70 (0.39-1.27)	13/13	0.91 (0.46-1.80)	0.92 (0.45-1.87)
	High	11/20	0.91 (0.42-1.96)	0.91 (0.42-1.97)			
Ethylene glycol	None	366/624	1.00	1.00			
	Low	6/17	0.56 (0.22-1.47)	0.54 (0.20-1.44)			
	High						
Diethylether	None				357/588	1.00	1.00
	Low				21/35	1.05 (0.59-1.86)	1.06 (0.59-1.90)
	High				8/22	0.59 (0.25-1.41)	0.58 (0.24-1.41)
**Chlorinated solvents**	None	313/519	1.00	1.00	375/622	1.00	1.00
	Low	27/66	0.65 (0.40-1.05)	0.62 (0.38-1.00)	11/23	0.86 (0.41-1.80)	0.87 (0.41-1.84)
	High	32/56	0.95 (0.59-1.52)	0.91 (0.56-1.48)			
Trichloroethylene	None	315/525	1.00	1.00	378/628	1.00	1.00
	Low	25/63	0.63 (0.38-1.03)	0.59 (0.35-0.97)	8/17	0.82 (0.35-1.95)	0.83 (0.35-1.99)
	High	32/53	1.00 (0.63-1.61)	0.98 (0.61-1.60)			
Perchloroethylene	None	362/631	1.00	1.00			
	Low	10/10	1.78 (0.72-4.38)	1.55 (0.60-3.99)			
	High						
Methylene chloride	None	360/622	1.00	1.00			
	Low	5/10	0.91 (0.30-2.74)	0.94 (0.33-2.72)			
	High	7/9	1.31 (0.48-3.58)	1.60 (0.59-4.31)			

Table S9 *continued*. Odds ratios and 95% confidence intervals for TGCT associated with parental occupational exposure to solvents in the year of the child's birth, when excluding cases not confirmed by pathology reports, TESTIS study, 2015-2018.

Solvents exposure		Paternal exposure			Maternal exposure		
		Ca/Co	Crude OR (95% CI) [a]	*Adjusted OR (95% CI)[a,b]*	Ca/Co	Crude OR (95% CI) [a]	*Adjusted OR (95% CI)[a,b]*
**Fuels & Petroleum-based solvents**	None	269/458	1.00	1.00	356/598	1.00	1.00
	Low	63/105	1.03 (0.72-1.48)	1.04 (0.72-1.50)	23/35	1.01 (0.58-1.75)	0.97 (0.55-1.70)
	High	40/78	0.84 (0.55-1.28)	0.78 (0.50-1.20)	7/12	0.99 (0.38-2.57)	0.90 (0.34-2.39)
Benzene	None	358/610	1.00	1.00			
	Low	6/16	0.71 (0.27-1.87)	0.59 (0.22-1.61)			
	High	8/15	0.93 (0.38-2.26)	0.93 (0.37-2.29)			
Automobile gasoline	None	352/598	1.00	1.00			
	Low	12/22	0.75 (0.36-1.59)	0.75 (0.35-1.61)			
	High	8/21	0.65 (0.28-1.50)	0.58 (0.24-1.39)			
Special petroleum products	None	359/623	1.00	1.00			
	Low<br>High	13/18	1.33 (0.63-2.80)	1.14 (0.53-2.44)			
White spirits	None	308/500	1.00	1.00	363/604	1.00	1.00
	Low	39/80	0.83 (0.55-1.26)	0.81 (0.53-1.24)	18/29	0.90 (0.48-1.66)	0.81 (0.43-1.51)
	High	25/61	0.67 (0.41-1.10)	0.70 (0.42-1.16)	5/12	0.74 (0.25-2.13)	0.93 (0.34-2.55)
Diesel, kerosene, fuel oil	None	321/565	1.00	1.00	379/637	1.00	1.00
	Low	42/50	1.48 (0.94-2.31)	1.52 (0.96-2.40)	7/8	1.59 (0.56-4.49)	1.47 (0.50-4.29)
	High	9/26	0.59 (0.27-1.30)	0.50 (0.22-1.13)			

Abbreviations - OR: odds ratio; 95% CI: confidence interval of 95%; Ca/Co: Cases/controls.

[a] Estimates obtained comparing TGCT cases to group A and group B controls combined.

[b] Estimates obtained comparing TGCT cases to group A and group B controls combined and adjusted for sibship size, being born from multiple pregnancy, personal history of testicular trauma, family history of TGCT and family history of cryptorchidism. Analysis was restricted to subjects with no missing data for the adjustment variables (N=12) and excluding cases not confirmed by pathology reports (N=43). If the "Low"/"High" categories have less than 5 cases or controls, the categories are grouped into "None" or "All" if the number of subjects was sufficient, otherwise the line is shaded.

Table S10. Odds ratios and 95% confidence intervals for TGCT associated with parental occupational exposure to solvents in the year of the child's birth, with an additional adjustment for age, TESTIS study, 2015-2018.

Solvents exposure		Paternal exposure		Maternal exposure	
		Ca/Co	OR (95% CI)[a,b]	Ca/Co	OR (95% CI) [a,b]
**Oxygenated solvents**	None	326/473	1.00	348/518	1.00
	Low	47/84	0.74 (0.49-1.12)	72/113	0.95 (0.67-1.34)
	High	40/84	0.70 (0.46-1.08)	5/14	0.46 (0.13-1.67)
Alcohols	None	361/539	1.00	352/524	1.00
	Low	25/51	0.77 (0.45-1.31)	73/121	0.88 (0.63-1.25)
	High	27/51	0.92 (0.56-1.53)		
Ketones and esters	None	381/577	1.00	410/619	1.00
	Low	20/44	0.62 (0.34-1.12)	15/13	0.88 (0.43-1.78)
	High	12/20	1.04 (0.48-2.22)		
Ethylene glycol	None	404/624	1.00		
	Low	9/17	0.52 (0.20-1.39)		
	High				
Diethylether	None			392/588	1.00
	Low			24/35	0.85 (0.48-1.50)
	High			9/22	0.82 (0.35-1.91)
**Chlorinated solvents**	None	344/519	1.00	413/622	1.00
	Low	31/66	0.60 (0.37-1.00)	12/23	0.87 (0.41-1.84)
	High	38/56	0.95 (0.59-1.53)		
Trichloroethylene	None	346/525	1.00	417/628	1.00
	Low	29/63	0.58 (0.35-0.96)	8/17	0.81 (0.34-1.92)
	High	38/53	1.01 (0.62-1.64)		
Perchloroethylene	None	403/631	1.00		
	Low	10/10	1.56 (0.61-3.97)		
	High				
Methylene chloride	None	399/622	1.00		
	Low	6/10	0.91 (0.30-2.74)		
	High	8/9	1.47 (0.53-4.07)		
**Fuels & Petroleum-based solvents**	None	295/458	1.00	392/598	1.00
	Low	72/105	0.99 (0.69-1.41)	25/35	1.02 (0.58-1.78)
	High	46/78	0.80 (0.52-1.23)	8/12	0.86 (0.33-2.24)
Benzene	None	396/610	1.00		
	Low	7/16	0.63 (0.23-1.70)		
	High	10/15	0.90 (0.37-2.21)		
Automobile gasoline	None	389/598	1.00		
	Low	14/22	1.04 (0.49-2.19)		
	High	10/21	0.56 (0.24-1.33)		
Special petroleum products	None	400/623	1.00		
	Low	13/18	1.16 (0.54-2.47)		
	High				
White spirits	None	336/500	1.00	399/604	1.00
	Low	47/80	0.79 (0.52-1.20)	20/29	0.95 (0.51-1.76)
	High	30/61	0.71 (0.43-1.17)	6/12	0.72 (0.25-2.10)
Diesel, kerosene, fuel oil	None	355/565	1.00	418/637	1.00
	Low	46/50	1.56 (0.99-2.44)	7/8	1.25 (0.43-3.62)
	High	12/26	0.46 (0.20-1.03)		

Abbreviations - OR: odds ratio; 95% CI: confidence interval of 95%; Ca/Co: Cases/controls.

[a] Estimates obtained comparing TGCT cases to group A and group B controls combined.

[b] Estimates obtained comparing TGCT cases to group A and group B controls combined and adjusted for sibship size, being born from multiple pregnancy, personal history of testicular trauma, family history of TGCT, family history of cryptorchidism and age at diagnosis (for cases)/age at inclusion (for controls). Analysis was restricted to subjects with no missing data for the adjustment variables (N=12) and excluding cases not confirmed by pathology reports (N=43). If the "Low"/"High" categories have less than 5 cases or controls, the categories are grouped into "None" or "All" if the number of subjects was sufficient, otherwise the line is shaded.

Table S11. Distribution of parental exposure average level of exposure for an 8-hour working day, by solvent and by class, TESTIS study, 2015-2018.

	**Fathers' level of exposure** (N=1054)				**Mothers' level of exposure** (N=1070)			
	**BZ** N (%)	**TCE** N (%)	**PCE** N (%)	**MC** N (%)	**BZ** N (%)	**TCE** N (%)	**PCE** N (%)	**MC** N (%)
Class 0	1006 (95.5)	871 (82.6)	1034 (98.1)	1021 (96.9)	1060 (99.1)	1045 (97.7)	1066 (99.6)	1055 (98.6)
Class 1	24 (2.3)	131 (12.4)	11 (1.0)	13 (1.2)	10 (0.9)	12 (1.1)	3 (0.3)	3 (0.3)
Class 2	3 (0.3)	42 (4.0)	1 (0.1)	13 (1.2)	0 (0.0)	11 (1.0)	0 (0.0)	0 (0.0)
Class 3	20 (1.9)	0 (0.0)	8 (0.8)	5 (0.5)	0 (0.0)	0 (0.0)	0 (0.0)	8 (0.7)
Class 4	1 (1.0)	10 (1.0)	0 (0.0)	2 (0.2)	0 (0.0)	2 (0.2)	1 (0.1)	4 (0.4)
**Max. value** (ppm)	1.5	37.5	18.8	26.3	0.1	56.3	28.1	28.1

Abbreviations – BZ: Benzene ; TCE: Trichloroethylene; PCE: Perchloroethylene ; MC: Methylene chloride ; I: Intensity; F: Frequency; Max. value: Maximum value.

For BZ, class 1 corresponds to exposure between 0,01 and 0,25 ppm for 8-hour working day; class 2 between 0,25 and 0,50 ppm; class 3 between 0,50 and 1 ppm; class 4: >1 ppm. For TCE, class 1 corresponds to exposure between 0,01 and 10 ppm for 8-hour working day; class 2 between 10 and 20 ppm; class 3 between 20 and 30 ppm; class 4: >30 ppm. For PCE and MC, class 1 corresponds to exposure between 0,01 and 10 ppm for 8-hour working day; class 2 between 10 and 15 ppm; class 3 between 15 and 20 ppm; class 4: >20 ppm. The average level for an 8-hour workday was obtained as the product of probability and intensity of exposure to the solvent for each job.
